# Quantitative Co-Expression of Proteins at the Single Cell Level – Application to a Multimeric FRET Sensor

**DOI:** 10.1371/journal.pone.0027321

**Published:** 2011-11-17

**Authors:** Joachim Goedhart, Laura van Weeren, Merel J.W. Adjobo-Hermans, Ies Elzenaar, Mark A. Hink, Theodorus W.J. Gadella

**Affiliations:** 1 Section of Molecular Cytology, van Leeuwenhoek Centre for Advanced Microscopy, Swammerdam Institute for Life Sciences, University of Amsterdam, Amsterdam, The Netherlands; 2 Department of Biochemistry, Nijmegen Centre for Molecular Life Sciences, Radboud University Nijmegen Medical Centre, Radboud University, Nijmegen, The Netherlands; Institut Européen de Chimie et Biologie, France

## Abstract

**Background:**

Co-expression of proteins is generally achieved by introducing two (or more) independent plasmids into cells, each driving the expression of a different protein of interest. However, the relative expression levels may vary strongly between individual cells and cannot be controlled. Ideally, co-expression occurs at a defined ratio, which is constant among cells. This feature is of particular importance for quantitative single cell studies, especially those employing bimolecular Förster Resonance Energy Transfer (FRET) sensors.

**Methodology/Principal Findings:**

Four co-expression strategies based on co-transfection, a dual promotor plasmid, an internal ribosome entry site (IRES) and a viral 2A peptide were selected. Co-expression of two spectrally separable fluorescent proteins in single living cells was quantified. It is demonstrated that the 2A peptide strategy can be used for robust equimolar co-expression, while the IRES sequence allows expression of two proteins at a ratio of approximately 3:1. Combined 2A and IRES elements were used for the construction of a single plasmid that drives expression of three individual proteins, which generates a FRET sensor for measuring heterotrimeric G-protein activation. The plasmid drives co-expression of donor and acceptor tagged subunits, with reduced heterogeneity, and can be used to measure G-protein activation in single living cells.

**Conclusions/Significance:**

Quantitative co-expression of two or more proteins can be achieved with little cell-to-cell variability. This finding enables reliable co-expression of donor and acceptor tagged proteins for FRET studies, which is of particular importance for the development of novel bimolecular sensors that can be expressed from single plasmid.

## Introduction

Genetically encoded Förster Resonance Energy Transfer (FRET) based biosensors have revealed novel insights in spatial and temporal aspects of protein interactions or conformations in a wide variety of cellular processes [1], [2]. These sensors often consist of two interacting proteins or a protein and an interacting domain sandwiched between a donor and an acceptor fluorophore. Changes in interaction or conformation lead to a FRET, which is quantified and used as a read-out. Unimolecular sensors are favored since (i) they are expressed from a single plasmid and (ii) the YFP over CFP ratio is constant among cells, simplifying quantification of FRET [3]. However, unimolecular sensors require the two interacting proteins or domains to be physically linked, which is not always possible due to structural constraints or post-translational modifications at the C- or N-terminus[4]. In such cases the two interacting proteins, fused to donor and acceptor fluorophores, need to be expressed separately. An advantage of bimolecular sensors is that the dynamic range is potentially larger, since the proteins are physically separated in absence of interaction and, hence, there is no baseline FRET in the non-interacting state [5], [6], [7].

To achieve co-expression of (fluorescent) proteins in a single cell, the proteins are typically expressed from separate plasmids, which are simply mixed in the transfection procedure. The main disadvantage of this approach is that the proteins are expressed at widely varying ratios and a subpopulation of cells only expresses one of the two constructs, which hampers FRET studies. Another drawback is that the development of stably expressing cells or organisms requires at least two independent transformation events, with hardly any control over the donor-to-acceptor ratio. To address these issues we set out to evaluate the performance of several strategies to co-express proteins reliably at a defined ratio in single living cells. We found that IRES and viral 2A peptides can be used to co-express proteins at a fixed ratio at the single cell level.

Subsequently, we employed these strategies to achieve expression of a multimolecular FRET sensor that measures the activation of a heterotrimeric G-protein complex from a single plasmid. The FRET sensor is composed of three proteins (CFP-tagged Gαq, Gβ and YFP-tagged Gγ), which were previously expressed using three separate plasmids [4]. Robust co-expression of CFP and YFP tagged subunits from a single plasmid was achieved and it was used for measuring G-protein activation in single living cells, with limited cell-to-cell variation in the FRET ratio.

## Results

**Figure 1:**
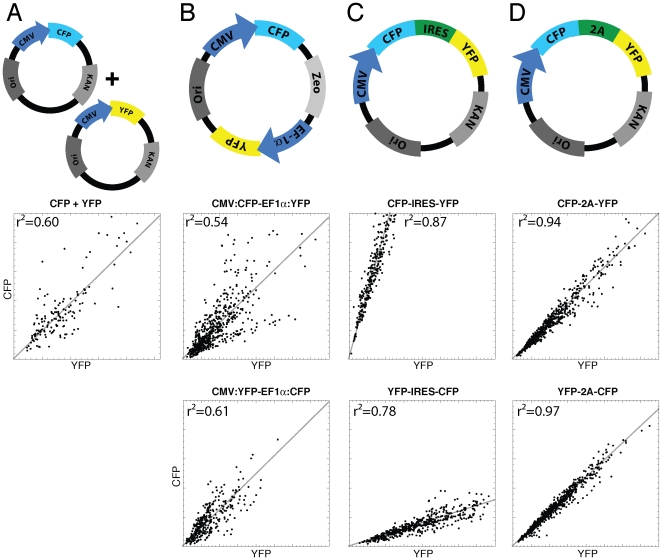
Characterization of different co-expression strategies by quantification of cyan fluorescent protein and yellow fluorescent protein fluorescence from single cells. Four different strategies for co-expression are analyzed: mixing two plasmids (A), two promoters on a single plasmid (B), an internal ribosome entry site (C) and a 2A viral cleavable peptide sequence (D). The upper row schematically depicts the used plasmids. The second and third row show the single cell based analysis of CFP versus YFP fluorescence (in arbitrary units) in the two possible orientations. The dots represent fluorescence intensity data from a single cell. The data set was fit with a linear line as a visual aid. The square of the correlation coefficient, r^2^, is indicated in the graph.

Several strategies allow proteins to be co-expressed at an (close to) equimolar ratio, as analyzed by biochemical assays on cell populations. Since it is unclear how these strategies perform in individual cells, we decided to co-express two almost identical reporter proteins CFP and YFP (98.6% identical at nucleotide level and 97.5% identical at protein level) using several strategies and evaluate their performance at the single cell level. First, ordinary mixing of equal amounts of plasmid encoding respectively the CFP variant mTurquoise[8] and the YFP variant mVenus(L68V) [9] was performed, followed by transfection. Quantification of fluorescence from single cells in the CFP and YFP channel showed marked heterogeneity in the CFP to YFP expression ratio (**figure 1A**). These results are in line with previous observations and it is suggested that the variation is caused by a limited number of plasmids that will finally end up in the nucleus [10].

We reasoned that expression of CFP and YFP from a single plasmid would yield cells that co-express the proteins at a constant ratio. First, a dual promoter plasmid designed to express two individual proteins was tested. Expression of CFP and YFP was driven by the CMV and EF-1alpha promoter, respectively. Analysis of the CFP versus YFP fluorescence indicated a striking heterogeneity, which was similar to that observed with the plasmid mixing strategy (**figure 1B**).

The IRES sequence has been used extensively to co-express two proteins from a single promoter. It has been known that the level of the protein translated from the IRES sequence is attenuated relative to the expression level of the upstream protein. The relative abundance of the two proteins depends on the IRES sequence used. The ECMV IRES was reported to be relatively efficient as analyzed both biochemically with antibodies and on living cells using fluorescence microscopy [11]. The ECMV IRES was therefore tested in a co-expression experiment. Co-expression of CFP and YFP was observed in all cells analyzed and the level of protein expressed from the IRES was reduced. Importantly, there was a strong correlation between CFP and YFP expression at the single cell level (**Figure 1C**).

Viral 2A peptides are peptide sequences of approximately 20 amino acids, which are employed by viruses to express two proteins at equal levels [12], [13]. The hypothesis is that the ribosome is stalled during translation, which leads to inefficient peptide bond formation. The ribosome continues and consequently two separate proteins are produced, together with a small fraction of fused protein. To investigate whether this strategy can be used to co-express fluorescent proteins we have fused CFP and YFP with a 2A peptide linker in the two possible orientations, i.e. CFP-2A-YFP and YFP-2A-CFP. As a control, a non-separable version was introduced (CFP-XX-YFP and YFP-XX-CFP). Co-expression analysis showed excellent correlation between CFP and YFP expression in single cells (**Figure 1D**), with no apparent dependence on the orientation of CFP and YFP.

The separation of the proteins was determined by quantification of the FRET efficiency by fluorescence lifetime imaging microscopy (FLIM). In case of fused protein, there should be efficient FRET between CFP and YFP, as indicated by a reduced excited state lifetime of CFP, while no FRET is expected when the CFP and YFP molecules are not connected. Indeed, we observed reduced lifetimes, in the non-separable controls, with a FRET efficiency of about 20%. The separable versions, CFP-2A-YFP and YFP-2A-CFP, did not exhibit a reduction in CFP lifetime, indicating no FRET (**Table 1**), which agrees with previous observations [13]. The absence of FRET indicates the efficient separation of CFP and YFP.

**Table 1:** FRET efficiency determined by FLIM of 2A constructs with mTurquoise as donor fluorescent protein.

Plasmid	n^1^	τ_ϕ_ [ns]^2^	τ_M_ [ns]^3^	E τ_ϕ_ [%]^4^	E τ_M_ [%]^4^
YFP-IRES-CFP	22	3.67±0.07	3.77±0.04	<1	<1
CFP-2A-YFP	22	3.64±0.04	3.83±0.02	2	<1
CFP-XX-YFP	16	2.91±0.04	3.28±0.04	21	14
YFP-2A-CFP	23	3.62±0.02	3.79±0.02	2	<1
YFP-XX-CFP	22	2.82±0.04	3.18±0.03	24	16

1 n number of cells from which the lifetime is calculated,

2 τ_ϕ_ average phase lifetime ± standard deviation,

3 τ_M_ average modulation lifetime ± standard deviation,

4 E average FRET efficiency calculated from τ_ϕ_ or τ_M_ according to (1-(τ_DA_/τ_D_))*100%, with τ_D_ values of 3.7 ns and 3.8 ns for mTurquoise phase and modulation lifetime [8].

Having established that both the IRES and the 2A viral peptide strategy can be used to express two proteins with a strong correlation between their respective expression levels, we determined the relative concentrations of the two proteins. Since it is rather difficult to do this accurately with fluorescence microscopy, we chose to employ FCS on cell extracts, as it is capable of quantifying the number of fluorescent molecules in a calibrated volume element. In case of FRET the counts per molecules of the donor will be decreased, but this will not affect the number of molecules which are detected in the volume element.

We detected stoichiometric expression of CFP and YFP when the non-separable CFP-XX-YFP and YFP-XX-CFP fusions were analyzed, suggesting complete maturation of both proteins. Subsequent analysis of CFP-2A-YFP and YFP-2A-CFP constructs revealed equal expression of CFP and YFP (**Table 2**). Cross-correlation analysis by FCCS [14] of CFP and YFP signals showed a substantial signal for the CFP-YFP non-separable control indicating presence of fusion protein, while no cross-correlation was detected in the 2A constructs and IRES constructs, indicating expression of separate proteins (data not shown).

**Table 2:** Results of the quantification of CFP and YFP from cell extracts by FCS.

Plasmid	n^1^	CFP:YFP^2^
CFP-2A-YFP	4	0.98±0.02
CFP-XX-YFP	4	0.93±0.03
YFP-2A-CFP	4	0.99±0.01
YFP-XX-CFP	4	1.04±0.03
YFP-IRES-CFP	4	0.43±0.01
CFP-IRES-YFP	4	3.11±0.06

1 n corresponds to the number of measurements from which the concentrations are calculated.

2 Average CFP to YFP ratio ± standard deviation, including error propagation for the standard deviation in individual concentrations and uncertainty of the detection volume sizes.

In case of FCS analysis of CFP and YFP expressed from plasmids with an IRES, there was a clear orientation dependence with a 2.5- to 3-fold higher cap-dependent expression of fluorescent protein relative to the downstream fluorescent protein, which is expressed from the IRES (**Table 2**). Together, our results show that 2A and IRES sequences can be used to co-express proteins at a defined ratio in single cells.

**Figure 2:**
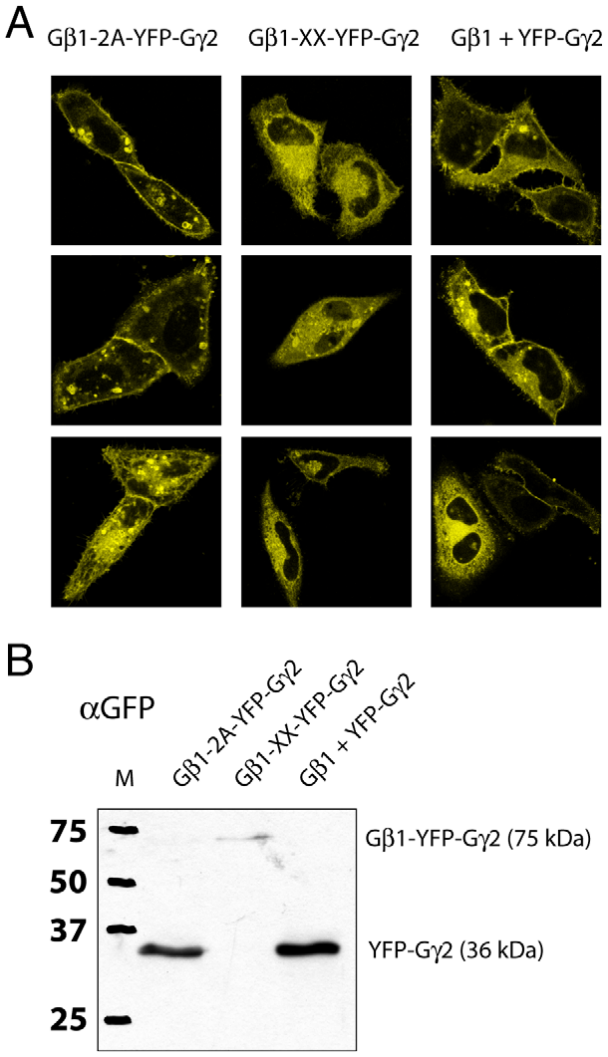
Analysis of Gβ1 and YFP-Gγ2 co-expression from a single plasmid or separate plasmids. (A) Three representative images of the localization of YFP-Gγ2 by confocal microscopy expressed from plasmid encoding Gβ1-2A-YFP-Gγ2, Gβ1-XX-YFP-Gγ2 or two separate plasmids, Gβ1+YFP-Gγ2. YFP-Gγ2 localizes to the plasma membrane and endomembranes in case of Gβ1-2A-YFP-Gγ2 expression and when Gβ1 and YFP-Gγ2 are co-expressed from a separate plasmids. The inseparable control Gβ1-XX-YFP-Gγ2 shows (dim) cytoplasmic and endomembrane fluorescence. The width of the images is 73 µm. (B) Western blotting with a GFP antibody confirms that YFP-Gγ2 is correctly expressed from the Gβ1-2A-YFP-Gγ2 plasmid and that non-separated product is not detectable. Protein expressed from Gβ1-XX-YFP-Gγ2 shows a faint band that corresponds to non-separated protein.

Heterotrimeric G proteins consist of three subunits Gα, Gβ and Gγ in a 1∶1∶1 complex [15]. Several groups, including ours, have shown that co-transfection of fluorescent tagged G-protein subunits from three separate plasmids driving expression of Gα, Gβ and Gγ yields a heterotrimeric G-protein complex. Moreover, the G-protein activation can be monitored in single cells by FRET ratio imaging [16], [17], [18], [19]. However, as observed in any co-transfection experiment, the CFP versus YFP fluorescence varies widely with a subpopulation expressing only either of the two fluorescent protein tagged subunits (**Figure S1**). To alleviate this limitation we examined whether it is possible to express the three proteins from a single plasmid using the aforementioned strategies. First the Gβ1 was linked via a 2A viral peptide to YFP-Gγ2. The CaaX box at the C-terminus of Gγ2 is essential for lipid modification and does not tolerate additional amino acids, preventing the use of the 2A sequence at this site. Therefore, we chose to construct a plasmid that expresses Gβ1-2A-YFP-Gγ2. Since the 2A linker peptide may introduce some non-separated product we compared the co-expression of Gβ1 and YFP-Gγ2 from separate plasmids and from plasmids expressing Gβ1-2A-YFP-Gγ2 and a non-separable control, Gβ1-XX-YFP-Gγ2. Localization by confocal microscopy (**Figure 2A**) and western blot (**Figure 2B**) showed that the expression of YFP-Gγ2 was indistinguishable when expression of separate plasmids was compared to expression from a single Gβ1-2A-YFP-Gγ2. Both expression strategies yielded a clear plasma membrane localization of YFP-Gγ2, besides some endomembrane labeling (**Figure 2A**). In contrast, the non-separable control localizes on endomembranes and no plasma membrane labeling is observed. Additionally, it is poorly expressed (data not shown), possibly due to rapid degradation. Furthermore, the western blot detects a faint high-molecular weight band in the sample that was transfected with the non-separable version Gβ1-XX-YFP-Gγ2 (**Figure 2B**). The molecular weight corresponds to the size of a protein consisting of Gβ1 fused to YFP-Gγ2, as expected from a non-cleavable 2A sequence. The low intensity agrees with the low amount of fluorescence observed in cells.

**Figure 3:**
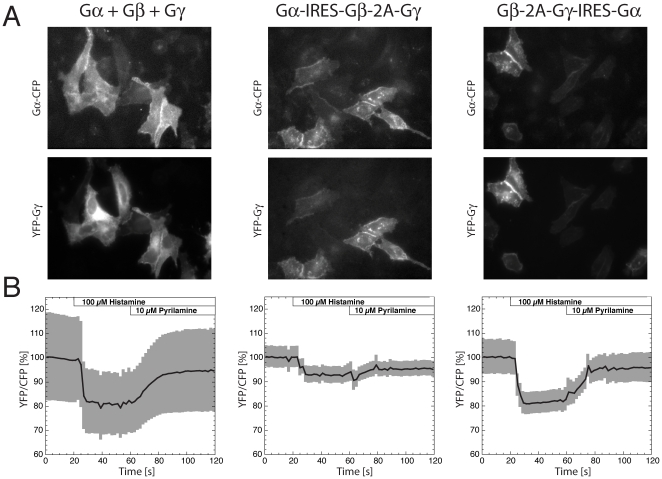
Characterization of a FRET sensor for monitoring G-protein activation expressed from a single plasmid or multiple plasmids. (A) Gαq-CFP, untagged Gβ1 and YFP-Gγ2 were expressed by either mixing three separate plasmids (Gα+Gβ+Gγ) or from a single plasmid in two orientations, Gα-IRES-Gβ-2A-Gγ and Gβ-2A-Gγ-IRES-Gα respectively. Fluorescence images of cells are shown depicting the Gαq-CFP and YFP-Gγ2 localization and expression levels. The width of the images is 177 µm. (B) FRET ratio-imaging (excitation of CFP and measuring the YFP over CFP intensity) data showing the time-course of an average ratio-change as a percentage (solid line) and s.e.m. (gray shading) of cells transfected with Gα+Gβ+Gγ (n = 13), Gα-IRES-Gβ-2A-Gγ (n = 12) and Gβ-2A-Gγ-IRES-Gα (n = 12). The average (± s.e.m.) initial YFP/CFP ratio that we determined are for Gα+Gβ+Gγ 2.1±0.4, for Gα-IRES-Gβ-2A-Gγ 0.76±0.03 and for Gβ-2A-Gγ-IRES-Gα, 1.5±0.1. HeLa cells were stimulated with 100 µM Histamine (t = 20s) and the response was reversed by adding the antagonist pyrilamine (t = 60 s).

Intact, unmodified C- and N-termini are essential for biofunctionality of Gα subunits [20] and therefore prohibits the use of a 2A viral peptide. Therefore, we chose to link the expression of Gαq-CFP via the IRES sequence to the Gβ1-2A-YFP-Gγ2 unit. We constructed two plasmids with different orientations, Gα-IRES-Gβ-2A-Gγ and Gβ-2A-Gγ-IRES-Gα. Qualitatively, we observed an improved correlation between the expression level of Gαq-CFP and YFP-Gγ2, for cells that expressed the tagged subunits from these plasmids, when compared to expression from separate plasmids (**figure 3A**). Moreover, both Gαq-CFP and YFP-Gγ2 were expressed in all cells and correctly localized to the plasma membrane. Quantitative analysis, in which YFP versus CFP fluorescence was measured from YFP-Gγ and Gαq-CFP respectively, reveals improved correlation for the single-plasmid systems. The highest correlation between CFP and YFP intensity (r^2^ = 0.5) was observed for the plasmid that expresses Gβ-2A-Gγ-IRES-Gα (**Figure S1**). It is of note that the CFP fluorescence intensity in this experiment is decreased by FRET, and that cellular autofluorescence contributes to both the CFP and the YFP channel, due to low expression of the G-protein subunits. Therefore, the measured intensities correlate with the amount of molecules, but can not be converted into numbers of molecules.

The formation of the heterotrimer was examined by quantification of the FRET efficiency by FLIM. FLIM of both constructs showed a reduced lifetime, indicating the formation of a heterotrimeric complex (**table 3**). The FRET efficiency of the Gβ-2A-Gγ-IRES-Gα construct was almost two-fold higher, when compared to the construct with the reverse orientation, Gα-IRES-Gβ-2A-Gγ. The FRET efficiency of Gβ-2A-Gγ-IRES-Gα based on the phase lifetime of 27% was similar to FRET efficiency that was reported before by transfecting the individual plasmids [4].

**Table 3:** FRET efficiency determined by FLIM of heterotrimers with mTurquoise as donor fluorescent protein.

Plasmid	n^1^	τ_ϕ_ [ns]^2^	τ_M_ [ns]^3^	E τ_ϕ_ [%]^4^	E τ_M_ [%]^4^
Gαq-mTurquoiseΔ6	18	3.6±0.1	3.8±0.1	<1	<1
Gα-IRES-Gβ-2A-Gγ	23	3.2±0.3	3.5±0.2	14	7
Gβ-2A-Gγ-IRES-Gα	22	2.7±0.2	3.1±0.2	27	19

1 n number of cells from which the lifetime is calculated,

2 τ_ϕ_ average phase lifetime ± standard deviation,

3 τ_M_ average modulation lifetime ± standard deviation,

4 E average FRET efficiency calculated from τ_ϕ_ or τ_M_ according to (1-(τ_DA_/τ_D_))*100%, with τ_D_ values of 3.7 ns and 3.8 ns for mTurquoise phase and modulation lifetime [8].

Previously, we have used the Gαq-CFP/YFP-Gγ2 FRET pair for ratiometric imaging to visualize heterotrimeric G-protein activation. When separate transfection of the subunits is employed, the initial YFP/CFP ratio varies widely between cells, due to variation in the relative expression level of Gαq-CFP versus YFP-Gγ2. To examine whether the YFP/CFP ratio is less variable in case of expression from a single plasmid we measured FRET ratios from cells transfected with Gα-IRES-Gβ-2A-Gγ or Gβ-2A-Gγ-IRES-Gα, and noticed a strongly reduced variation as inferred from a reduced standard error of the mean (**Figure 3B**).

We attempted to measure the relative expression of Gαq-CFP and YFP-Gγ2 by FCS on cell extracts, but the fluorescence levels were too close to background. Future studies will address the stoichiometry of the G-protein complex in single living cells.

Subsequently, we monitored G-protein activation by monitoring the FRET ratio and stimulation of cells with the H1 receptor agonist histamine. The measurements with subunits expressed from multiple plasmids show the largest variation in ratio, while the single plasmid strategies show reduced heterogeneity (**figure 3B** **).** The average ratio-change was close to 20% for the Gα+Gβ+Gγ and Gβ-2A-Gγ-IRES-Gα sample, while it was only around 10% for the Gα-IRES-Gβ-2A-Gγ sample. The higher ratio-change of Gβ-2A-Gγ-IRES-Gα and higher initial FRET efficiency (**Table 3**) are most likely due to a more favorable donor/acceptor ratio. To conclude, we demonstrate that expression of a multimeric FRET sensor for heterotrimeric G-protein activation from a single plasmid reduces heterogeneity in the FRET ratio between cells, while retaining a high dynamic range.

## Discussion

Here, we demonstrate that co-transfection of two independent plasmids results in co-expression of two proteins with high cell-to-cell variation in the relative expression level. A similar heterogeneity was observed when the two fluorescent proteins were expressed from a single vector with two promotors. It is of note that alternative vectors, not tested in this study, are commercially available for co-expressing two proteins. This reports provides a method for testing their performance at the single cell level. The observed variation is problematic for quantitative single cell studies, especially those employing bimolecular or multimeric FRET sensors.

Two different approaches, the viral 2A peptide and the IRES sequence present a solution. The 2A cleavable peptide is an elegant way to co-express multiple proteins at equimolar levels from a single transcript. At least four proteins have been co-expressed using this 2A strategy to induce pluripotent stem cells [21], although it remains to be shown whether all four proteins are expressed at equimolar levels in single cells. A possible drawback of 2A peptides is a minor fraction of uncleaved protein, which is undesired and may give rise to false-positive FRET, although we did not observe non-separated product when Gβ1 and Gγ2 were co-expressed from the plasmid encoding Gβ1-2A-YFP-Gγ2. It is possible that the amount of uncleaved protein product is smaller in case of tagged proteins, since these are amenable to degradation and generally less stable than untagged fluorescent proteins. Another issue that should be considered is the N- or C-terminal modification by the inserted peptide.

The IRES yields two proteins that are completely physically separated and not modified at C- or N-terminus. As the upstream protein is expressed at an approximately 3-fold higher level than the protein downstream of the IRES, it is possible to express donor and acceptor at either a 1:3 or 3:1 ratio. We demonstrate that this feature can be used to tune the FRET efficiency of the heterotrimer. By combining IRES and 2A sequences we have constructed a multimeric FRET sensor for G-protein activation expressed from a single plasmid. Although we show that we can control the relative expression levels of Gαq-CFP and YFP-Gγ2, it is of note that we do not have control over protein degradation rates and post translational modifications, which is generally beyond the control of the experimenter. The single plasmid expressing Gβ-2A-Gγ-IRES-Gα shows the best improvement, with a reduced heterogeneity in FRET ratio, when compared to expression from three individual plasmids, in combination with a good FRET response (20% change) range upon G-protein activation.

Together, our study shows that the 2A and IRES based strategies hold great potential for quantitative co-expression of multiple proteins with little cell-to-cell variability. These findings will be useful for the development of bimolecular genetically encoded FRET sensors expressed from a single plasmid. These type of approaches require somewhat more complex cloning strategies, but will simplify the transformation of cells or organisms for stable expression of multiple proteins. In general, robust co-expression approaches will be important tools for quantitative single cell studies and for multiplexing applications in which as many as possible (tagged) proteins need to be expressed and visualized simultaneously.

## Methods

### Plasmid Construction

For the co-expression analysis we used two bright visible fluorescent proteins (VFPs), mTurquoise [8] and mVenus(L68V) [9], which are indicated as CFP and YFP respectively.

The pBUD-CE4 vector was from Invitrogen (Breda, The Netherlands) and uses the Zeocin resistance marker. VFPs were cut using HindIII and XbaI from the VFP-N1 vector (Clontech) and inserted behind the CMV promoter of pBUD, which was cut with the same enzymes.

To insert a VFP downstream of EF1a, the VFPs were amplified using the fw-EF 5′- GGAAGATCTCCACCATGGTGAGCAAGG-3′ and the rv-EF primer 5′- GGAAGATCTGTCGCGGCCGCTTTACTTG-3′ with a VFP-N1 plasmid as template.

The product was cut with BglII and inserted into pBud cut with BglII. Two constructs were made in this way; CMV:CFP-EF1a:YFP and CMV:YFP-EF1a:CFP.

Construction of 2A plasmids was described previously [8]. A 2A linker is inserted between the coding sequences of the VFPs, encoding the peptide EGRGSLLTCGDVEENPGPGS. Two plasmids are constructed CFP-2A-YFP and YFP-2A-CFP. A mutated non-cleavable linker encodes EGRGSLLTCGDVEENAAPGS and two plasmids based on this linker are denoted as CFP-XX-YFP and YFP-XX-CFP.

The pPRIG-IRES vector [11] which carries the ECMV IRES sequence was obtained from Patrick Martin. The IRES sequence was amplified by PCR using the fw-IRES 5′- CTAGCTAGCGCCACCATGGAGATCTGGGCCCCTATAGTGTCAC-3′ and rv-IRES primer 5′- CGCGGATCCGGTTGTGGCCATATTATC-3′. A Clontech-C1 vector and the amplified IRES were cut using the restriction enzymes NheI and BamHI and ligated. The VFPs were cut from the pBUD vector using BglII and ligated upstream of the IRES and cut from the 2A plasmid vector using BamHI and ligated downstream of the IRES. Two constructs were made CFP-IRES-YFP and YFP-IRES-CFP.

Gαq-mTurquoiseΔ6, Gβ1 and YFP-Gγ2 were described previously [4] and used as components for a single plasmid expressing these three proteins. Using PCR-based cloning the plasmids expressing Gβ1-2A-YFP-Gγ2 and Gβ1-XX-YFP-Gγ2 were made. Two plasmids were subsequently constructed encoding either Gαq-mTurquoiseΔ6-IRES-Gβ1-2A-YFP-Gγ2 (Gα-IRES-Gβ-2A-Gγ) or Gβ1-2A-YFP-Gγ2-IRES-Gαq- mTurquoiseΔ6 (Gβ-2A-Gγ-IRES-Gα). The sequences and detailed construction procedures of these plasmids are available upon request.

### Cell culture and transfection

HeLa cells were obtained from the American Tissue Culture Collection (ATCC; Manassas, VA, USA) and cultured in DMEM+Glutamax (Invitrogen, #61965), 10% FBS, Penicillin (100 U/ml) and Streptomycin (100 µg/ml). Cells were transfected using 1-2 µl lipofectamine (Invitrogen), 0.5 µg plasmid DNA and 50 µl OptiMEM per 35 mm dish holding a 24 mm Ø #1 coverslip.

### Western Blot

Two days after transfection, cells from a single well of a 6-well plate were lysed by addition of 200 µl lysis buffer (PBS, 1% Triton X-100, 0.5% sodium deoxycholate 0.1% sodium dodecyl sulphate) and harvested by scraping. After spinning, 15 µl of the supernatant was used for SDS-PAGE. Immunolabeling was performed with 1∶2000 AntiGFP-serum (Invitrogen, A6455) and 1∶10,000 Goat anti-Rabbit IgG(H&L)-HRP Conjugate (Biorad, 170-6516) as a secondary antibody. Detection was performed with the Amersham Western Blotting Detection System (GE Healthcare RPN2132) and Hyperfilm ECL (28906838).

### Fluorescence imaging

Coverslips with cells were mounted in an Attofluor cell chamber (Invitrogen) and submerged in microscopy medium (20 mM HEPES (pH = 7.4), 137 mM NaCl, 5.4 mM KCl, 1.8 mM CaCl_2_, 0.8 mM MgCl_2_ and 20 mM glucose). Fluorescence imaging experiments were performed on a Zeiss 200M inverted fluorescence microscope using a Zeiss Plan-Neofluar 40×/1.30 Ph3 oil objective. Excitation light from a Cairn Xenon Arc lamp was selected by a monochromator (Cairn Research). For cyan fluorescent protein, 420 nm excitation light (slit 30 nm) was used and in case of yellow fluorescent proteins, 500 nm excitation light (slit 30 nm) was applied. Additional filtering of was done with CFP excitation filter E460SP (375-460) and YFP excitation filter E520SP (375–520). The dichroic mirror 455DCLP and emission filter BP470/30 were used for CFP and a dichroic mirror 515DCXR and emission filter BP535/30 were used for YFP fluorescence. Metamorph software was used for controlling the equipment. Exposure times were typically 100 ms for the CFP channel and 50 ms for the YFP channel. FRET ratio imaging was performed at 37°C as described before on at least 3 different samples [4].

FRET ratio imaging data were processed using ImageJ (http://rsbweb.nih.gov/ij/) by quantifying the average fluorescence intensity of individual cells in the CFP and the YFP channel and subtracting the background obtained from a region of interest without cells. These intensity data were used to calculate the YFP/CFP ratio. The ratio time-traces from at least 12 cells were not normalized but directly averaged and the standard error of the mean was calculated. The data was subsequently scaled to display the FRET ratio change as a percentage. This procedure was used to depict the heterogeneity of the ratios that we measured and is somewhat different to the processing procedure that is normally performed, in which heterogeneity and folding/maturation of the fluorescent proteins is compensated for by normalizing the individual CFP and YFP traces [4], [22], [23].

FLIM was performed as described before [4] at room temperature.

### Image Correction and Analysis

Fluorescence images for quantitative co-expression analysis were background corrected and subsequently corrected for shading using a homogenous fluorescent plastic slide (Chroma). Fluorescence intensity of single cells was quantified by drawing an ROI and measuring average fluorescence intensity in both CFP and YFP channels (in arbitray units). All corrections and analyses were performed using the ImageJ software with ObjectJ plug-in and macros. Graphs and fits of the average CFP versus YFP intensity of individual cells were made with KaleidaGraph. The correlation coefficient was determined using Excel (RSQ function).

### FCS

Cell extracts were freshly prepared from single wells of a 6-well plate. Transfected cells were washed two times with cold PBS. Cells were lysed with cold 300 µl PBS+1% (v/v) Triton X-100. After centrifugation (5 min. at 16,000 g), 200 µl supernatant was collected and the extracts were diluted 10–20x with PBS+0.01% Triton X-100.

Measurements were performed in glass-bottomed 96 wells plates (Whatman), placed on top of an inverted Fluoview 1000 laser scanning microscope (Olympus) equipped with a water immersed 60x UPLSApo objective (NA 1.2). The light of a 440 nm pulsing laser diode (Picoquant), operated at 20 MHz, was combined with the 514 nm line of a continuous wave Ar^+^ laser (Melles-Griot), using a polarizing beam cube in the excitation path. Via a 440/514/594 main dichroic mirror (Chroma) the emission light was guided through a size adjustable pinhole in the Olympus confocal detection box towards the fibre output channel. The optical fibre was coupled to a custom-made detection box (Picoquant) containing two avalanche photodiode detectors (MPD). An LP515 dichroic mirror (Semrock) was placed in the beam path to split the emission light and 475/42 (CFP) and 534/30 (YFP) emission filters (Semrock) were placed in front of the detectors. The photon arrival times were recorded by a Picoharp 300 unit (Picoquant).

Before correlating the detector signals, the raw data traces were time-gated in SymPhoTime 5.1.3 software (Picoquant) to prevent cross-talk of the dyes. Photons arriving in the CFP channel within 25 ns of the 440 nm excitation pulse were considered to be the true CFP signal, while the photons arriving in the YFP channel 25–50 ns after the 440 nm excitation pulse are contributing to the YFP signal. The signals were autocorrelated and the resulting curves were analysed with the standard triplet-diffusion model [24] to retrieve particle numbers. Control experiments were performed using solutions of Atto425 (D = 410 m^2^.s^−1^) and Alexa 488 (D = 400 m^2^.s^-1^) to calibrate the size and overlap of the CFP, YFP detection volumes. The obtained particle numbers were corrected for background signal [24].

## Supporting Information

Figure S1 **Quantitative co-expression analysis of YFP-Gγ2 versus Gαq-CFP.** The results of three different plasmids are shown, Gα+Gβ+Gγ (r^2^ = 0.004), Gα-IRES-Gβ-2A-Gγ (r^2^ = 0.03) and Gβ-2A-Gγ-IRES-Gα (r^2^ = 0.5). The r^2^ values between brackets represent the square of the correlation coefficient. The dots represent fluorescence intensity data from a single cell. The data set was fit with a linear line as a visual aid.
(TIF)
