# Supplementary figures and images for: Quantitative Co-Expression of Proteins at the Single Cell Level – Application to a Multimeric FRET Sensor
Source: PLoS One. 2011 Nov 17;6(11):e27321. doi: 10.1371/journal.pone.0027321 (PMC3219669; doi:10.1371/journal.pone.0027321)

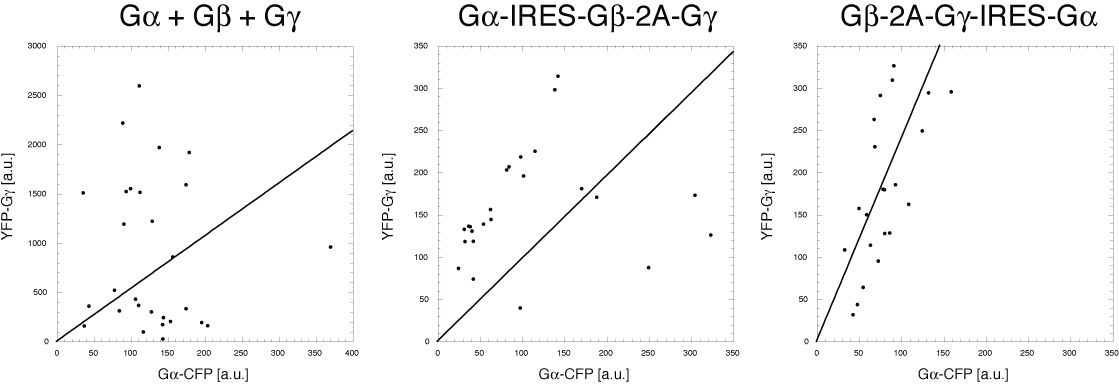

Supplement: Figure S1 — Quantitative co-expression analysis of YFP-Gγ2 versus Gαq-CFP. The results of three different plasmids are shown, Gα+Gβ+Gγ (r2 = 0.004), Gα-IRES-Gβ-2A-Gγ (r2 = 0.03) and Gβ-2A-Gγ-IRES-Gα (r2 = 0.5). The r2 values between brackets represent the square of the correlation coefficient. The dots represent fluorescence intensity data from a single cell. The data set was fit with a linear line as a visual aid. (TIF) [file pone.0027321.s001.tif]
